# Treatment-Resistant Fulminant Septic Shock: A Case of Multidrug-Resistant Streptococcus pneumoniae Bacteremia in an Unvaccinated Intravenous Drug User

**DOI:** 10.7759/cureus.82185

**Published:** 2025-04-13

**Authors:** Elene Saribekovi, Elene Pachkoria, Tamar Didbaridze, Tamar Megrelishvili, Nino Gogokhia, Ia Mikadze, Levan Ratiani

**Affiliations:** 1 Department of Infectious Diseases, Tbilisi State Medical University, Tbilisi, GEO; 2 Department of Infectious Diseases, First University Clinic of Tbilisi State Medical University, Tbilisi, GEO; 3 Department of Microbiology, First University Clinic of Tbilisi State Medical University, Tbilisi, GEO; 4 Department of Laboratory Medicine, First University Clinic of Tbilisi State Medical University, Tbilisi, GEO; 5 Department of Anesthesiology, First University Clinic of Tbilisi State Medical University, Tbilisi, GEO

**Keywords:** antibiotic resistance, intravenous drug use, pneumococcal vaccination, septic shock, streptococcus pneumoniae bacteremia

## Abstract

This case report presents *Streptococcus pneumoniae* bacteremia in a 60-year-old male with a history of intravenous drug use (IDU), presenting with acute respiratory distress syndrome (ARDS). The patient experienced fever, malaise and myalgia for about a week. Chest imaging revealed diffuse bilateral infiltrates, and laboratory tests showed elevated inflammatory markers. His condition deteriorated abruptly, rapidly progressing to respiratory failure, shock, and ultimately death. Both sputum and blood cultures confirmed *Streptococcus pneumoniae* infection and revealed resistance to commonly used antibiotics, including ceftriaxone, azithromycin, and levofloxacin. Despite appropriate antibiotic therapy, the infection could not be controlled, and the patient’s condition deteriorated rapidly; he died on the fifth day of hospitalization due to multi-organ failure. The case underscores the challenges of managing sepsis and ARDS in IDU patients. Additionally, it emphasizes the growing issue of antibiotic resistance and importance of primary prevention, including vaccination, as a key strategy to reduce the incidence and severity of infections in IDUs.

## Introduction

Sepsis is a life-threatening condition that occurs when the body’s response to infection triggers widespread inflammation, leading to tissue damage, organ failure, and potentially death. It remains one of the leading causes of hospital morbidity and mortality worldwide, with the United States alone reporting approximately 1.7 million cases annually, resulting in approximately 350,000 deaths each year [1]. Sepsis is particularly dangerous in populations with underlying risk factors, such as intravenous drug use (IDU), which increases susceptibility to bacteremia, endocarditis, and other infectious diseases, often causing or contributing to the development of sepsis.

One of the primary causes of sepsis-related acute respiratory distress syndrome (ARDS) is pneumonia, with *Streptococcus pneumoniae* (pneumococcus) being the leading pathogen responsible for community-acquired pneumonia. Pneumococcal pneumonia can result in significant morbidity, and sepsis-associated ARDS carries a mortality rate of 30-40% [2].

A major global health concern is the increasing antibiotic resistance in *Streptococcus pneumoniae*, particularly against commonly used β-lactams and macrolides. This resistance is fueled by the overuse and misuse of antibiotics. The horizontal transmission of antibiotic resistance genes further complicates the clinical management of these infections [3].

Vaccination against *S. pneumoniae *is a preventive measure that can significantly reduce the incidence and severity of these infections. Currently, pneumococcal conjugate vaccines (PCVs), such as PCV13, PCV15, and PCV20, along with the Pneumococcal polysaccharide vaccine (PPSV23), are key strategies for prevention. Pneumococcal vaccination is recommended for infants and young children, adults aged 50 years or older, and individuals with specific risk factors such as chronic medical conditions, asplenia, and immunocompromised states [4].

This case underscores the critical importance of pneumococcal vaccination in high-risk groups, including individuals with a history of intravenous drug use. Moreover, the early symptoms of infections, particularly in this risk group, can often be nonspecific and unclear, which complicates timely diagnosis and intervention. The patient presented with a severe *Streptococcus pneumoniae* infection, which was resistant to commonly used antibiotics such as ceftriaxone and azithromycin, highlighting the growing challenge of treating pneumococcal infections. Despite sensitivity, there was no response to piperacillin-tazobactam and vancomycin, further complicating the treatment approach.

## Case presentation

A 60-year-old male with a history of heavy alcohol and intravenous drug use, but no other known chronic medical conditions, was admitted to the emergency department with fever, myalgia, and malaise lasting for about a week. However, in the 24 hours prior to admission, his condition rapidly worsened, resulting in respiratory failure, which ultimately required hospitalization. Upon admission, the patient was tachypneic (respiratory rate 36 breaths per minute), with a blood pressure of 142/82 mmHg, heart rate of 144 beats per minute, and a temperature of 37.5°C. He appeared cyanotic with an oxygen saturation (SPO₂) of 41%, which improved to 85-88% on high-flow oxygen and non-invasive ventilation (NIV/CPAP) (Table 1).

**Table 1:** Dynamics of Clinical Decline: From Admission to Death SPO₂: Oxygen Saturation NIV: Non-Invasive Ventilation CPAP: Continuous Positive Airway Pressure P-SIMV: Pressure-Synchronized Intermittent Mandatory Ventilation

Day	SPO₂ (%)	Blood Pressure (mmHg)	Temperature (°C)	Ventilation
1	85-88	142/82	37.5	NIV/CPAP
2	75	130/80	38	P-SIMV
3	80	115/75	39.2	P-SIMV
4	88	90/53	39.8	P-SIMV
5	75	85/50	40	P-SIMV

On auscultation, the patient exhibited decreased breath sounds, fine crackles, and wheezing diffusely across both lungs. His clinical condition rapidly deteriorated, requiring orotracheal intubation and mechanical ventilation.

The initial laboratory investigations were significant for a slightly decreased white blood cell count and elevated C-reactive protein (Table 2).

**Table 2:** Blood Analysis Results on the Day of Admission WBC: White Blood Cell Neu: Neutrophile Lymph: Lymphocyte CRP: C-Reactive Protein CREA: Creatinine

Laboratory Parameter	Value	Reference Range
WBC (×10⁹/L)	3.26	4.0–11.0
Neu (×10⁹/L)	2.42	2.0-7.0
Lymph (×10⁹/L)	0.76	1-5
Bands (×10⁹/L)	0.08	0.01-0.2
CRP (mg/L)	>200	<5
CREA (µmol/L)	62	54.00–104.00

**Figure 1:**
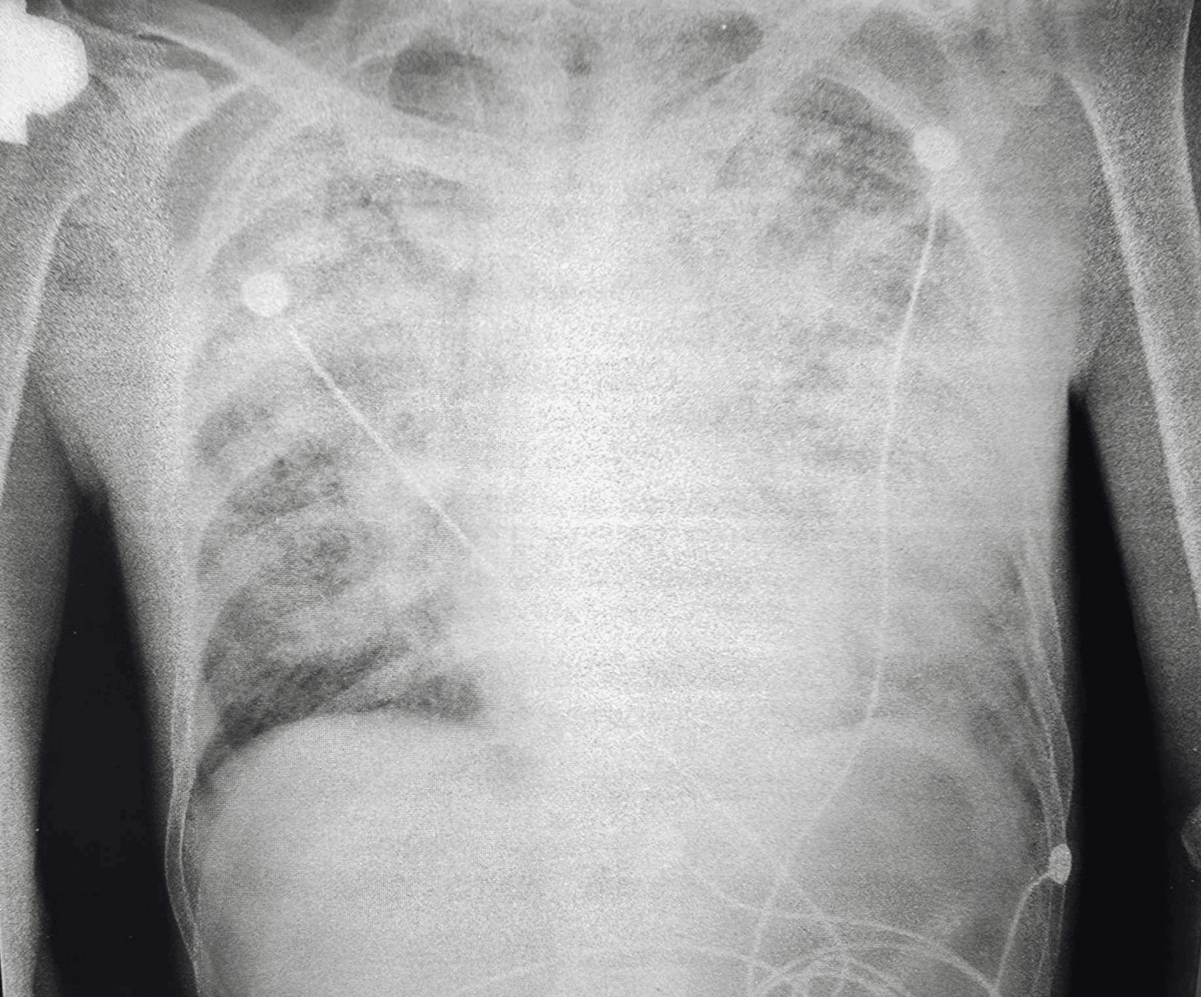
Chest X-ray findings: Significant reduction in lung aeration with extensive diffuse bilateral infiltrates involving both lung fields, more pronounced on the left side

Chest X-ray findings showed a significant decrease in lung aeration with confluent bilateral infiltrates affecting both lung fields, more pronounced on the left side, suggesting pneumonia with ARDS (Figure 1). The partial pressure of oxygen in arterial blood/fraction of inspired oxygen (P/F) ratio of 65 indicated severe impairment in oxygenation, consistent with severe ARDS. Echocardiography revealed moderate mitral valve regurgitation (+2/4), mild pulmonary hypertension, with a pulmonary artery systolic pressure (PASP) of 46 mmHg (normal range: 15-30 mmHg) and no vegetations were detected.

Empirical antibiotic therapy with piperacillin-tazobactam and vancomycin, along with supportive care, was initiated. On day three of admission, blood and sputum cultures identified *Streptococcus pneumoniae*, which showed the same antibiotic susceptibility pattern in both samples (Table 3). Based on the antibiotic susceptibility profile, meropenem was added to the antibiotic therapy regimen.

**Table 3:** Antibiotic Susceptibility Test Results

Antibiotic	Susceptibility
Piperacillin-tazobactam	Sensitive
Imipenem	Sensitive
Meropenem	Sensitive
Vancomycin	Sensitive
Cotrimoxazole	Sensitive
Oxacillin	Sensitive
Linezolid	Sensitive
Teicoplanin	Sensitive
Ceftriaxone	Resistant
Levofloxacin	Resistant
Moxifloxacin	Resistant
Azithromycin	Resistant
Clindamycin	Resistant
Tetracycline	Resistant

**Figure 2:**
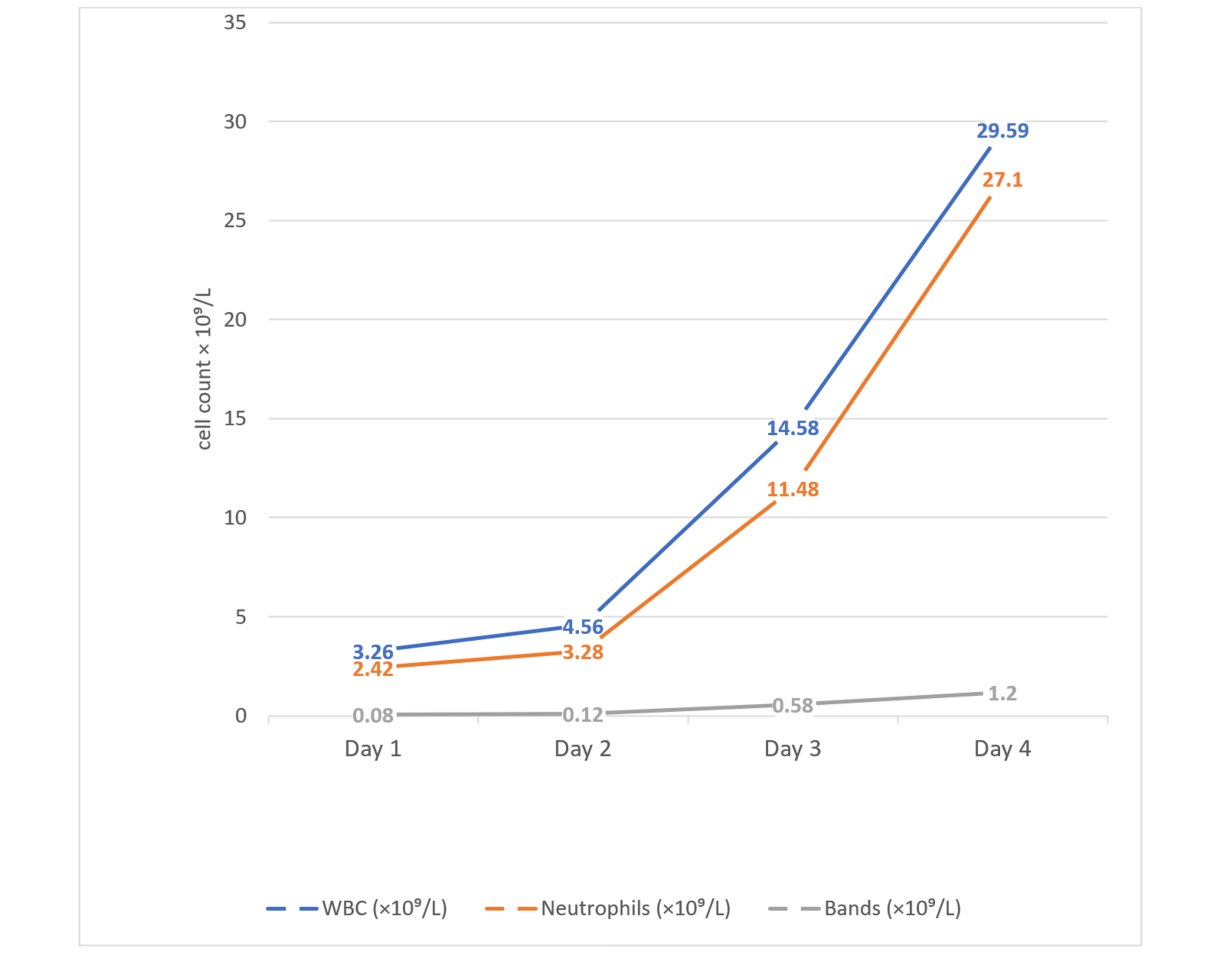
Dynamics of white blood cell count, neutrophil count, and bands from admission to day four of hospitalization, exhibiting sudden escalating leukocytosis WBC: White Blood Cell

Despite aggressive management, the patient's condition deteriorated on day four (Table 1), with a Glasgow Coma Scale of 4, and he remained hypoxic despite maximal mechanical ventilation. Laboratory findings showed significant decline, including creatinine level of 418.5 µmol/L (Table 4) and prominent neutrophilia with elevated bands (Figure 2). On day five, he died due to multi-organ failure resulting from septic shock.

**Table 4:** Blood Analysis Results on the Fourth Day of Admission WBC: White Blood Cell Neu: Neutrophile Lymph: Lymphocyte CRP: C-Reactive Protein CREA: Creatinine

Laboratory Parameter	Value	Reference Range
WBC (×10⁹/L)	29.59	4.0–11.0
Neu (×10⁹/L)	27.1	2.0-7.0
Lymph (×10⁹/L)	2.04	1-5
Bands (×10⁹/L)	1.2	0.01-0.2
CRP (mg/L)	>200	<5
CREA (µmol/L)	418	54.00–104.00

## Discussion

This case describes a 60-year-old male with a history of IDU, who initially presented with sepsis and ARDS. Despite the use of appropriate empirical antibiotics and intensive care, the patient ultimately experienced multi-organ failure and died on the fifth day of admission.

One of the main factors contributing to the patient's rapid deterioration was the delayed admission. The patient reported symptoms for a week before seeking medical care, during which the infection likely progressed significantly. The positive sputum and blood cultures identified *Streptococcus pneumoniae*. While intravenous drug use raised concerns for a bloodstream infection, there were no signs of injection site infection or pneumococcal endocarditis (PE), which is rare, accounting for less than 3% of infective endocarditis cases [5]. Instead, the chest X-ray revealed bilateral diffuse infiltrates, more pronounced on the left side (Figure 1), suggesting pneumonia complicated by sepsis and ARDS. The identification of the same bacterium in both sputum and blood cultures, along with identical antibiotic susceptibility patterns, confirms this route of infection.

Heavy alcohol and intravenous drug use may have contributed to aspiration pneumonia, as altered consciousness in these patients can lead to aspiration of oropharyngeal flora. While aspiration was once thought to primarily involve oral anaerobes, both community- and hospital-acquired pneumonias are now recognized to result from aspiration of virulent pathogens, including *S. pneumoniae* [6]. Pneumonia should always be suspected in ARDS with sepsis, even in the absence of strong radiographic evidence, as the airspace opacities caused by ARDS on the chest X-ray can obscure the presence of associated pneumonia [7].

Another key concern highlighted by this case was the antibiotic resistance profile of the isolated *Streptococcus pneumoniae* strain. In the Intensive Care Unit, pneumonia is commonly treated empirically with a beta-lactam plus a macrolide [8]. The organism showed resistance to several commonly used antibiotics, including ceftriaxone, azithromycin, levofloxacin and moxifloxacin (Table 3). This resistance profile is concerning, particularly in the context of IDU, where frequent or inappropriate antibiotic use can increase the risk of developing resistant infections.

Although piperacillin-tazobactam and vancomycin were included in the initial treatment regimen, they did not yield a positive effect, as the infection process severely progressed. Considering the challenges of antibiotic resistance and the progression dynamics, vaccination against *S. pneumoniae* becomes increasingly important. Pneumococcal vaccination (PCVs and PPSV23) should be prioritized in IDUs, along with other recommended immunizations such as hepatitis A, hepatitis B, influenza, COVID-19, and Tdap, due to often incomplete or unknown immunization histories in this population. Prioritizing vaccination programs for IDUs can help reduce the incidence of severe infections and limit the need for antibiotics.

## Conclusions

This case report highlights the critical challenges in managing treatment-resistant fulminant septic shock caused by *Streptococcus pneumoniae* in an unvaccinated IDU. This case underscores the alarming reality of the growing issue of multidrug resistance, which can significantly impair treatment effectiveness and contribute to poor outcomes. Rapid deterioration despite appropriate therapy highlights the need for primary prevention strategies. Therefore, this case reinforces the critical importance of vaccination as an essential measure to reduce the burden of severe pneumococcal infections, minimizing the need for intensive, and sometimes ineffective, antibiotic treatment.
